# Society Meetings

**Published:** 1894-06

**Authors:** 


### Society Meetings.

The Association of Erie Railway Surgeons will hold its next meeting at the International hotel, Niagara Falls, July 18, 1894, under the presidency of Dr. C. B. Kibler, of Corry, Pa. Drs. Outten, of St. Louis, Murdoch, of Pittsburg and J. B. Murphy, of Chicago, are expected to attend the meeting. Special rates will be extended to surgeons and ladies for both hotel and carriages. In order that all members may attend, it is important that they ask their division superintendent for transportation at least thirty days before the meeting.

The Michigan State Medical Society, at its recent annual meeting, elected the following named officers to serve for the ensuing year: President, H. O. Walker, Detroit; first vice-president, Victor C.

Vaughan, Ann Arbor; second vice-president, C. H. White, Reed City; third vice-president, Nina Logue, Adrian; fourth vice-president, C. H. McKain, Vicksburg; secretary, C. W. Hitchcock, Detroit; treasurer, William G. Henry, Detroit; judicial council, Fleming Carrow, Ann Arbor; Frank Garber, Muskegon; E. L. Shurly, Detroit.

The American Association of Obstetricians and Gynecologists will hold its seventh annual meeting at Toronto, Ont., Wednesday, Thursday and Friday, September 19, 20, 21, 1894. All physicians interested in the association, or the nature of its work, are cordially invited to attend the meeting. By order of the executive council.

The Medical Society of the County of Erie will hold its regular semi-annual meeting in the Academy of Medicine rooms, Market Arcade, Main street, Buffalo, Tuesday, June 12, 1894, under the presidency of Dr. William H. Gail, of East Aurora. The program will be issued soon.

